# Perspectives and Management of Atypical Asthma in Chinese Specialists and Primary Care Practitioners—A Nationwide Questionnaire Survey

**DOI:** 10.3389/fmed.2021.727381

**Published:** 2021-10-21

**Authors:** Huaqiong Huang, Wen Hua, Ruchong Chen, Yue Hu, Songmin Ying, Chunhua Chi, Min Zhang, Kewu Huang, Huiguo Liu, Huahao Shen, Kefang Lai

**Affiliations:** ^1^Key Laboratory of Respiratory Disease of Zhejiang Province, Department of Respiratory and Critical Care Medicine, Second Affiliated Hospital of Zhejiang University School of Medicine, Hangzhou, China; ^2^State Key Laboratory of Respiratory Disease, National Clinical Research Center for Respiratory Disease, Guangzhou Institute of Respiratory Health, First Affiliated Hospital of Guangzhou Medical University, Guangzhou, China; ^3^Department of General Practice, Peking University First Hospital, Beijing, China; ^4^Department of Respiratory and Critical Care Medicine, Shanghai General Hospital, Shanghai Jiao Tong University School of Medicine, Shanghai, China; ^5^Department of Pulmonary and Critical Care Medicine, Beijing Chao-Yang Hospital, Capital Medical University, Beijing, China; ^6^Key Laboratory of Pulmonary Diseases of Health Ministry, Department of Respiratory and Critical Care Medicine, Tongji Hospital, Tongji Medical College, Huazhong University of Science and Technology, Wuhan, China

**Keywords:** atypical asthma, surveys and questionnaires, clinical practice, WeChat, specialists and primary care practitioners

## Abstract

**Background and objective:** To evaluate the awareness/knowledge and clinical practice for the treatment of atypical asthma among respiratory specialists and primary care practitioners (PCPs) in China.

**Methods:** A total number of 1,997 physicians participated in the survey *via* WeChat. The questionnaire included six main items: physician demographic characteristics, awareness, diagnosis, medical prescription, assessment/education, and proposal.

**Results:** Cough variant asthma (CVA) was recognized by 97.51% of physicians (1,166 respiratory specialists and 799 PCPs), followed by chest tightness variant asthma (CTVA, 83.72%) and occult asthma (73.54%). Specialists were more likely to follow diagnostic recommendations than PCPs (*P* < 0.01); however, 34.15% of physicians reported the utility of bronchodilation tests, airway provocation tests, and peak expiratory flow monitoring. A total of 91.70% and 92.01% of physicians prescribed inhaled corticosteroids (ICS) or ICS plus long-acting beta-agonists (LABA) for CVA and CTVA, respectively. Physicians prescribed an ICS or ICS/LABA for 4 (2–8) or 8 (4–12) weeks for CVA and 4 (2–8) or 5 (4–12) weeks for CTVA, and the prescription durations were significantly shorter for PCPs than for specialists (*P* < 0.01). Further, 52.42% and 35.78% reported good control of CVA and CTVA, respectively, with significantly lower control rates for PCPs than for specialists (*P* < 0.01). Additionally, specialists exhibited better assessment and educational habits than PCPs.

**Conclusion:** While atypical asthma was identified by most specialists and PCPs, there remains a gap between management in real clinical practice and guideline recommendations, especially for PCPs. Further training of PCPs and clinical studies of atypical asthma are required to improve practice.

## Introduction

Asthma is a global health problem that affects ~334 million people worldwide, with an expected increase to 400 million worldwide by 2025, imposing a substantial burden on the healthcare system (1, 2). In China, the prevalence of asthma among adults is 4.2% (3). Classic asthma presents with symptoms of wheezing, breathlessness, and cough. Apart from the typical symptoms, asthma may manifest as various clinical phenotypes. In contrast to classic asthma, atypical forms of asthma, such as cough variant asthma (CVA) and chest tightness variant asthma (CTVA), present with symptoms of either cough or chest tightness without wheezing.

Three types of atypical asthma have been identified and described in the past half a century. In 1979, Corrao et al. first reported CVA, with cough as the only or main symptom (4); in 1992, the Zhong et al. team reported occult asthma (5); and in 2013, Shen et al. reported CTVA, with chest tightness as the only or main symptom (6). These types of atypical asthma have been listed in asthma guideline in China (7, 8). Many subsequent studies have found that atypical asthma has a pathobiological basis, namely, eosinophilic inflammation, and airway hyperresponsiveness similar to that of classic asthma (9, 10). Inhaled corticosteroids (ICS) or a combination of ICS and long-acting beta-agonists (ICS/LABA) resulted in a good response in patients with these asthma types compared with those with classic asthma (11, 12). These studies have aided physicians in understanding atypical asthma, reducing the rate of misdiagnosis and improving treatment outcomes. However, there are still many unknowns and challenges regarding atypical asthma.

It has often been reported that atypical asthma is underdiagnosed or misdiagnosed clinically (4, 6). Promisingly, some global and Chinese guidelines on asthma or chronic cough have added guidance for the management of atypical asthma (13–16). According to guidelines, for example, establishing a diagnosis depends on symptoms; objective tests; a good treatment response; and the exclusion of alternative diagnoses (13, 14). Such a complex process requires extensive physician knowledge/awareness and specialized on-site equipment, both of which can be a challenge for front-line primary care practitioners (PCPs). Moreover, studies on atypical asthma are scarce, and the sample sizes are small (12, 17–20).

To date, no previous study has reported the status of the management of atypical asthma. We hope to accurately understand atypical asthma awareness and practices to aid in the development of management strategies. The present survey was conducted in China and aimed to evaluate the level of atypical asthma knowledge and self-reported management practices by specialists and PCPs.

## Methods

### Study Design

The online survey was conducted in 25 provinces in China between Jan 16th and Feb 4th, 2021. A total of 1,997 physicians were invited to participate in the survey by the Asthma Group of the Respiratory Branch of the Chinese Medical Association. Among them, 1,187 specialists came from tier 3 or 2 hospitals and 810 PCPs from primary medical care facilities, respectively.

### Methods

The questionnaire was developed by the authors. It was designed to assess physician characteristics, physician knowledge/awareness, diagnosis, treatment practices, evaluation and management of atypical asthma. The main questions, excluding the questions regarding demographic characteristics, are listed in supporting (Supplementary Table E1). Online questionnaires were sent and returned *via* WeChat.

The diagnostic criteria for CVA, CTVA and occult asthma were defined according to the 2020 Chinese asthma guidelines (7) and 2015 Chinese cough guidelines (21), as shown in Table 1.

**Table 1:** Diagnostic Criteria of CVA and CTVA.

CVA	1. Cough is the only or main symptom, without wheezing, shortness of breath, and other symptoms and signs of typical asthma.
	2. Examination to support reversible airflow limitation.
	3. Except for other diseases that cause cough.
	4. It is effective to treat disease according to treatment for typical asthma.
CTVA	1. Chest tightness is the only or main symptom, without wheezing, shortness of breath, and other symptoms and signs of typical asthma.
	2. Examination to support reversible airflow limitation.
	3. Except for other diseases that cause chest tightness.
	4. It is effective to treat disease according to treatment for typical asthma.

### Statistical Analysis

All data were analyzed using SPSS 19.0 software. Continuous variables are expressed as means (SDs) for data with a normal distribution and as medians (IQRs) for data with a non-normal distribution, while classification variables analyzed with the chi square test are expressed as a numbers (percentages). *P* < 0.05 indicates statistical significance.

## Results

### Demographic Characteristics

The demographic characteristics of the providers are shown in Table 2. A total of 1,166 specialists (completion rate, 98.23%, 453 males and 713 females) and 799 PCPs (completion rate, 98.64%, 319 males and 480 females) were filled in the questionnaire completely and included in analysis.

**Table 2:** The demographic characteristics of the survey.

	**Total**		**Specialized hospitals**		**Primary medical care**		**χ^**2**^ value**	***P*-value**
*N*	1,965	100%	1,166	59.34%	799	40.66%		
Age								
20–30 years	200	10.18%	59	3.01%	141	7.18%	108.86	<0.0001
30–40 years	808	41.12%	544	27.69%	264	13.44%		
40–50 years	675	34.35%	372	18.94%	303	15.42%		
50–60 years	270	13.74%	184	9.37%	86	4.38%		
>60 years	12	0.61%	7	0.36%	5	0.25%		
Male	772	39.29%	453	23.06%	319	16.23%	0.2293	0.632
Female	1,193	60.71%	713	36.28%	480	24.43%		
Residents	319	16.23%	0	0.00%	319	16.23%	592.54	<0.0001
Attending	811	41.27%	524	26.66%	287	14.61%		
Deputy chief physician	483	24.58%	348	17.71%	135	6.87%		
Chief physician	352	17.91%	294	14.97%	58	2.95%		
Working years < 10	536	27.28%	358	18.22%	178	9.06%	16.9665	<0.0001
Working years > 10	1,429	72.72%	808	41.12%	621	31.60%		
College	271	13.79%	7	0.35%	264	13.44%	419.67	<0.0001
Bachelor degree or above	1,694	86.21%	1,159	58.98%	535	27.23%		

### Knowledge/Awareness of Atypical Asthma

**Figure 1:**
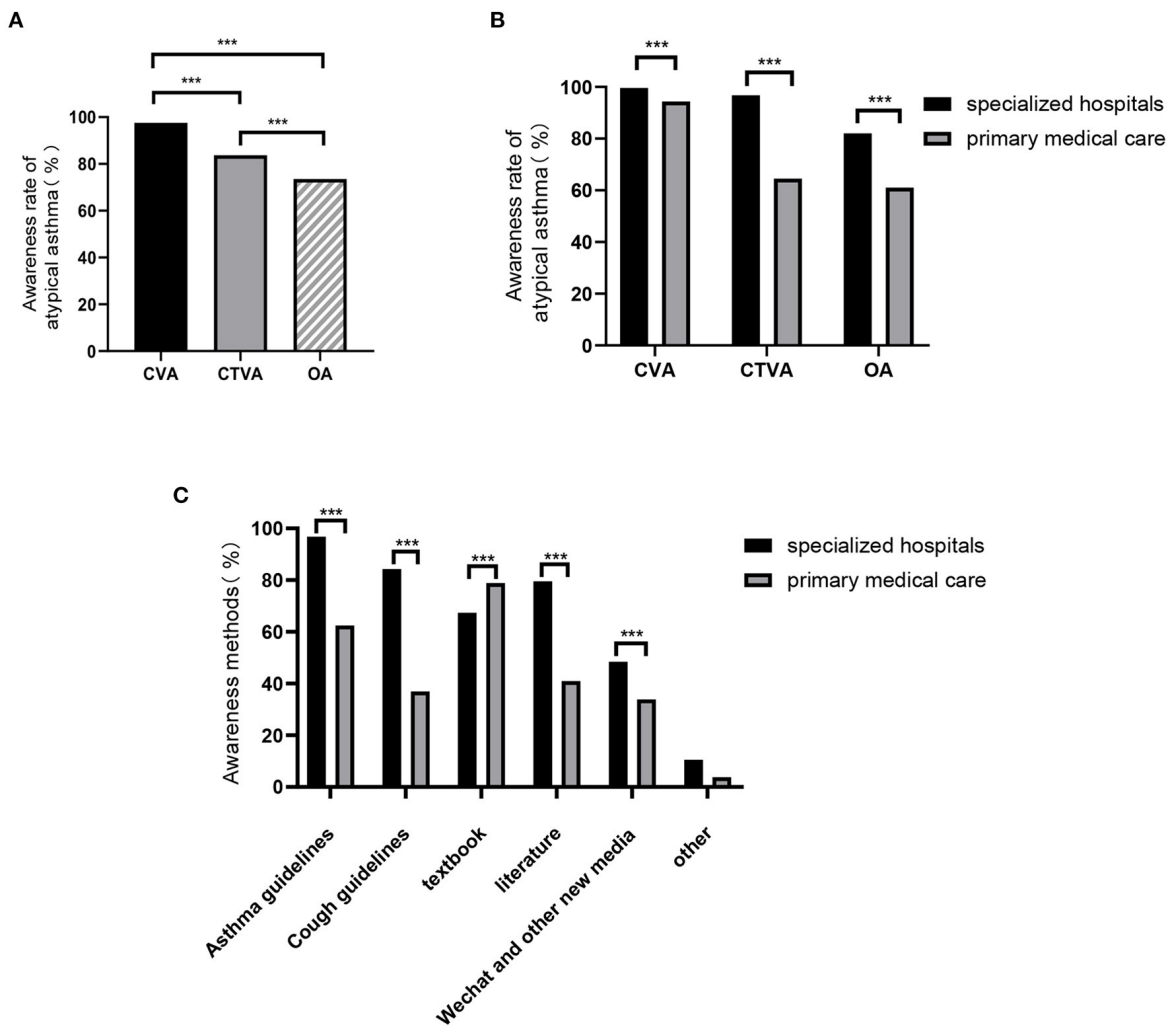
Knowledge/awareness of atypical asthma. **(A)** Awareness of atypical asthma, including CVA, CTVA, and OA, among physicians in both specialized hospitals and primary medical care facilities. Awareness rates of atypical asthma **(B)** and Awareness methods of atypical asthma **(C)** between physicians in specialized hospitals and primary medical care facilities. CVA, cough variant asthma; CTVA, chest tightness variant asthma; OA, occult asthma. ***P < 0.001.

Of the physicians who indicated that they were aware of CVA, CTVA and occult asthma were reported by 97.51%, 83.72%, and 73.54% of the physicians, respectively. CVA was the most well-known, followed by CTVA and occult asthma (*P* < 0.001, Figure 1A).

Overall, the proportion of specialists who were aware of CVA was higher than that of PCPs (99.66% vs. 94.37%, *P* < 0.001). This pattern was also observed for CTVA (96.83% vs. 64.58%, *P* < 0.001) and occult asthma (82.08% vs. 61.08%, *P* < 0.001, Figure 1B).

The most common way by which specialists acquired knowledge of atypical asthma was through guidelines, while that for PCPs was through textbooks (Figure 1C). Articles, WeChat and other resources were also methods noted by physicians.

### Diagnosis of CVA and CTVA

**Figure 2:**
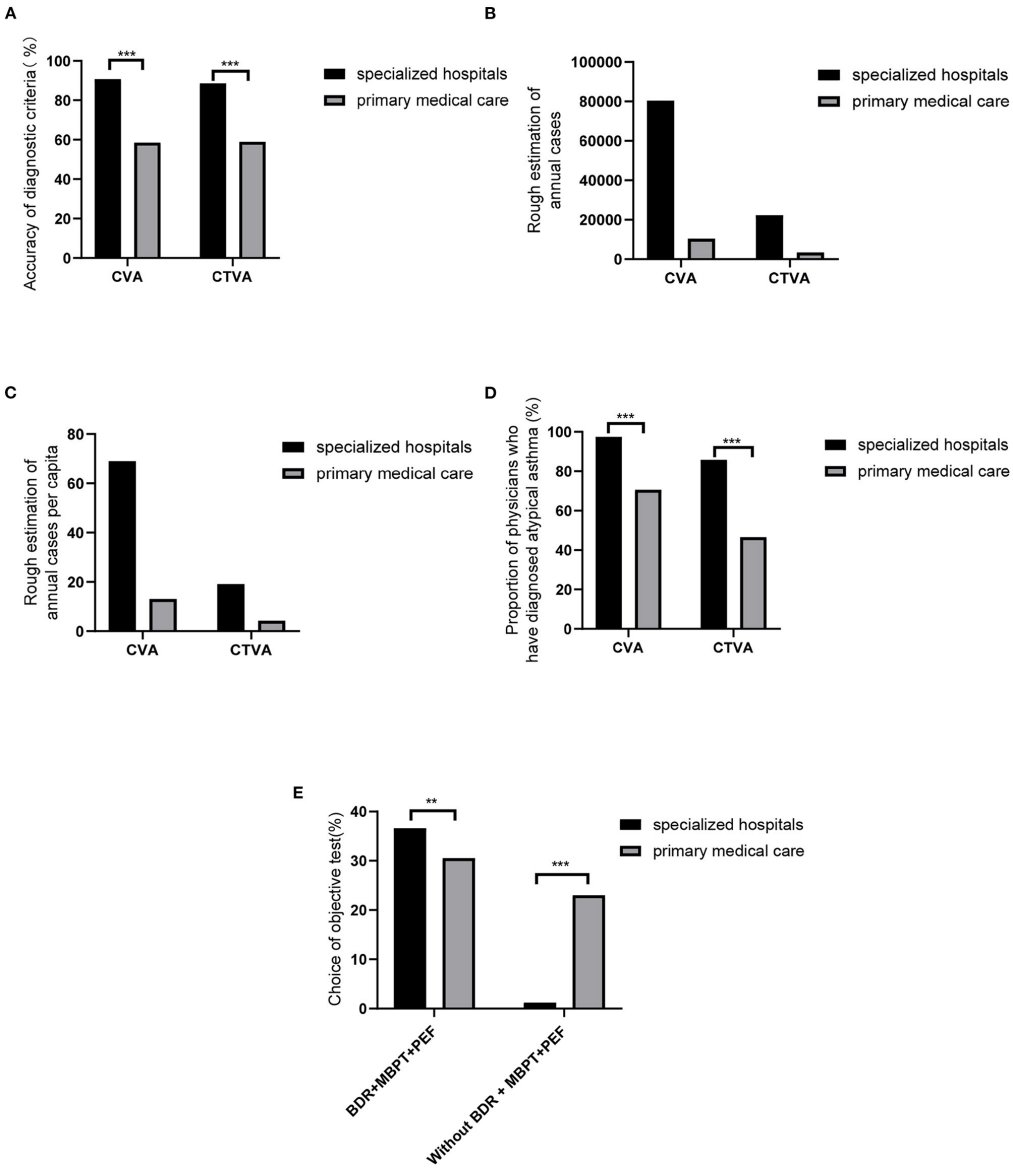
Diagnosis of CVA and CTVA. Accuracy of diagnostic criteria **(A)**, Rough estimation of annual cases **(B)**, Rough estimation of annual cases per capita **(C)**, Proportion of physicians who have diagnosed atypical asthma **(D)**, and Choice of objective test **(E)** in specialized hospitals and primary medical care facilities. BDR, bronchodilation tests; MBPT, methacholine challenge; PEF, peak expiratory flow. ^**^P < 0.01, ^***^P < 0.001.

The aim of Q6–Q7 in supporting Supplementary Table E1 was to determine agreement with or adherence of specialists and PCPs to the diagnostic criteria in the guidelines. The correct answer met all four options recommended in the 2020 Chinese asthma guidelines and 2015 Chinese cough guidelines. For CVA, 77.71% of the physicians' answers concurred with the diagnostic criteria. Only 58.6% of the PCPs responded with adequate answers for CVA diagnosis; this was significantly lower than that of specialists (58.57% vs. 90.82%, *P* < 0.001, Figure 2A). Similarly, in CTVA, a majority of physicians (76.54%) answered the survey correctly. Good knowledge of the diagnostic criteria was observed among specialists, and the knowledge level was significantly higher than that of PCPs (88.59% vs. 58.95%, *P* < 0.001, Figure 2A).

Physicians were asked “how many cases of CVA or CTVA did you diagnosis per week.” Based on these questions, self-reported cases by physicians in a year were accounted. Of the physicians surveyed, 1,166 specialists and 799 PCPs reported that ~80,397 and 10,392 patients were diagnosed with CVA annually (Figure 2B), and the average annual per capita numbers of diagnosed patients were 68.95 and 13.00, respectively (Figure 2C). Among them, 97.43% of specialists had diagnosed CVA. However, the ratios were significantly lower for PCPs (97.43% vs. 70.59%, *P* < 0.01, Figure 2D).

A total of 22,251 patients per year were diagnosed with CTVA by specialists, while only 3,367 patients were diagnosed with CTVA by PCPs (Figure 2B). In addition, the average annual per capita numbers of patients diagnosed by specialists and PCPs were 19.08 and 4.21, respectively (Figure 2C). Compared with PCPs, specialists had a higher rate of CTVA diagnosis (85.76% vs. 46.56%, *P* < 0.001, Figure 2D).

Bronchodilation tests, methacholine challenge or PEF are recommended identically in the diagnosis of atypical asthma. Therefore, the questionnaire setup utilized multiple-choice questions (Q8–Q12). However, only 34.15% of the physicians chose all three items (36.62% of specialists vs. 30.54% of PCPs); Meanwhile, as many as 23.03% of PCPs did not choose any of the three examination methods, while only 1.20% of specialists did (Figure 2E).

### Medications Prescribed for CVA and CTVA Treatment

**Figure 3:**
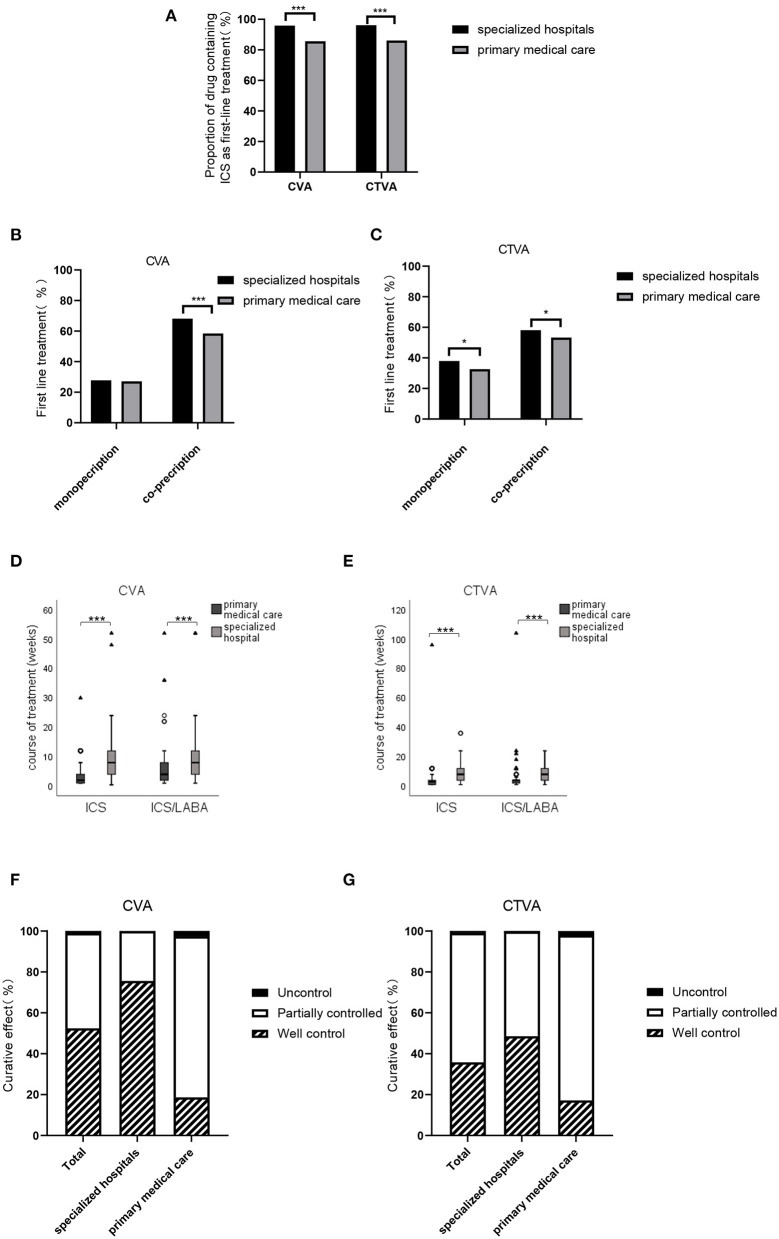
Medications prescribed for CVA and CTVA. **(A)** Proportion of physicians who prescribed an ICS as a first-line treatment for patients with CVA or CTVA in specialized hospitals and primary medical care facilities. **(B)** Proportion of physicians who prescribed an ICS or ICS+LABA as monotherapy or combined with other drugs as a first-line treatment for CVA in specialized hospitals and primary medical care facilities. **(C)** Proportion of physicians who prescribed ICS or ICS+LABA as monotherapy or combined with other drugs as a first-line treatment for patients with CTVA in specialized hospitals and primary medical care facilities. **(D)** Course of ICS or ICS+LABA treatment in patients with CVA in specialized hospitals and primary medical care facilities. Date are presented as medians [IQRs]. **(E)** Course of ICS or ICS+LABA treatment in patients with CTVA in specialized hospitals and primary medical care facilities. **(F)** Rates of well-controlled, partially controlled and uncontrolled CVA in all facilities, specialized hospitals and primary medical care facilities. **(G)** Rates of well-controlled, partially controlled and uncontrolled CTVA in all facilities, specialized hospitals and primary medical care facilities. ^*^P < 0.05, ^***^P < 0.001.

The first-line treatments for CVA and CTVA are shown in Figure 3A. For CVA, 91.70% of the physicians responded that they prescribed medications that included an ICS or an ICS/LABA as a first-line treatment. Specialists prescribed an ICS or an ICS/LABA at a higher rate than PCPs (95.88% vs. 85.61%, *P* < 0.001). Fewer physicians used ICS or ICS/LABA alone (27.79% of specialists and 27.16% of PCPs); a majority of physicians prescribed other drugs in combination with ICS or ICS/LABA in both specialists and PCPs (68.10% and 58.45%, respectively; *P* < 0.001) (Figure 3B). Co-prescribed medications included leukotriene receptor antagonists (LTRAs), theophylline, compound methoxamine, antimicrobial drugs, oral corticosteroids (OCSs), albuterol, and traditional Chinese medicines.

For CTVA, 92.01% of the physicians indicated that drugs containing an ICS or ICS/LABA were first-line treatments. Specialists prescribed significantly more ICS or ICS/LABA than PCPs (96.14% vs. 85.98%, *P* < 0.001). Similar to CVA, for CTVA, an ICS or an ICS/LABA was mostly used in combination with other drugs. In addition, several specialists and PCPs preferred to co-prescribed medication for first-line treatment of CTVA (Figure 3C).

The median course [IQR] of ICS and ICS/LABA treatment prescribed by physicians for CVA was 4 (2–8) and 8 (4–12) weeks, respectively. Patients who were diagnosed with CVA by specialists had longer ICS and ICS/LABA treatment courses, while those who were diagnosed by PCPs had a shorter therapy duration [median (IQR) 7 (4–10.5) weeks vs. 2 (1–4) weeks, *P* < 0.001; 8 (4–12) weeks vs. 4 (2–8) weeks, *P* < 0.001, respectively] (Figure 3D).

For the treatment of CTVA, ICS, and ICS/LABA were prescribed by physicians for a median (IQR) of 4 (2–8) weeks and 5 (4–12) weeks, respectively. The ICS and ICS/LABA reported by specialists were also significantly longer than PCPs [median (IQR) 6 (4–8.5) weeks vs. 3 (1–4) weeks, *P* < 0.001; 8 (4–12) weeks vs. 4 (2–4) weeks, *P* < 0.001, respectively] (Figure 3E).

Regarding the control level, well control and partial control were achieved in 98.88% of CVA case (Figure 3F) and 98.98% of CTVA case (Figure 3G). Among the control group, well control was achieved in 52.45% of CVA and 35.78% of CTVA (Table 3). Of patients under the care of specialists, well-controlled, partially controlled, and uncontrolled CVA was 75.56%, 24.36%, and 0.09%, respectively, and the corresponding CTVA was 48.54%, 51.20%, and 0.26%, respectively (Table 3). Compared with those in specialized hospitals, the rates of control of CVA and CTVA were lower in primary care facilities.

**Table 3:** Control level of CVA and CTVA.

		**Total**	**Specialized hospitals**	**Primary medical care**	*z* value^*^	***P*-value**
CVA	Well control	1,030 (52.45%)	881 (75.56%)	149 (18.65%)	24.72	<0.0001
	Partially controlled	913 (46.46%)	284 (24.36%)	629 (78.72%)		
	Uncontrol	22 (1.12%)	1 (0.09%)	21 (2.63%)		
CTVA	Well control	703 (35.78%)	566 (48.54%)	137 (17.15%)	14.54	<0.0001
	Partially controlled	1,242 (63.21%)	597 (51.2%)	645 (80.73%)		
	Uncontrol	20 (1.02%)	3 (0.26%)	17 (2.13%)		

* *Mann-Whiteney test*.

### Education and Management of CVA and CTVA

**Figure 4:**
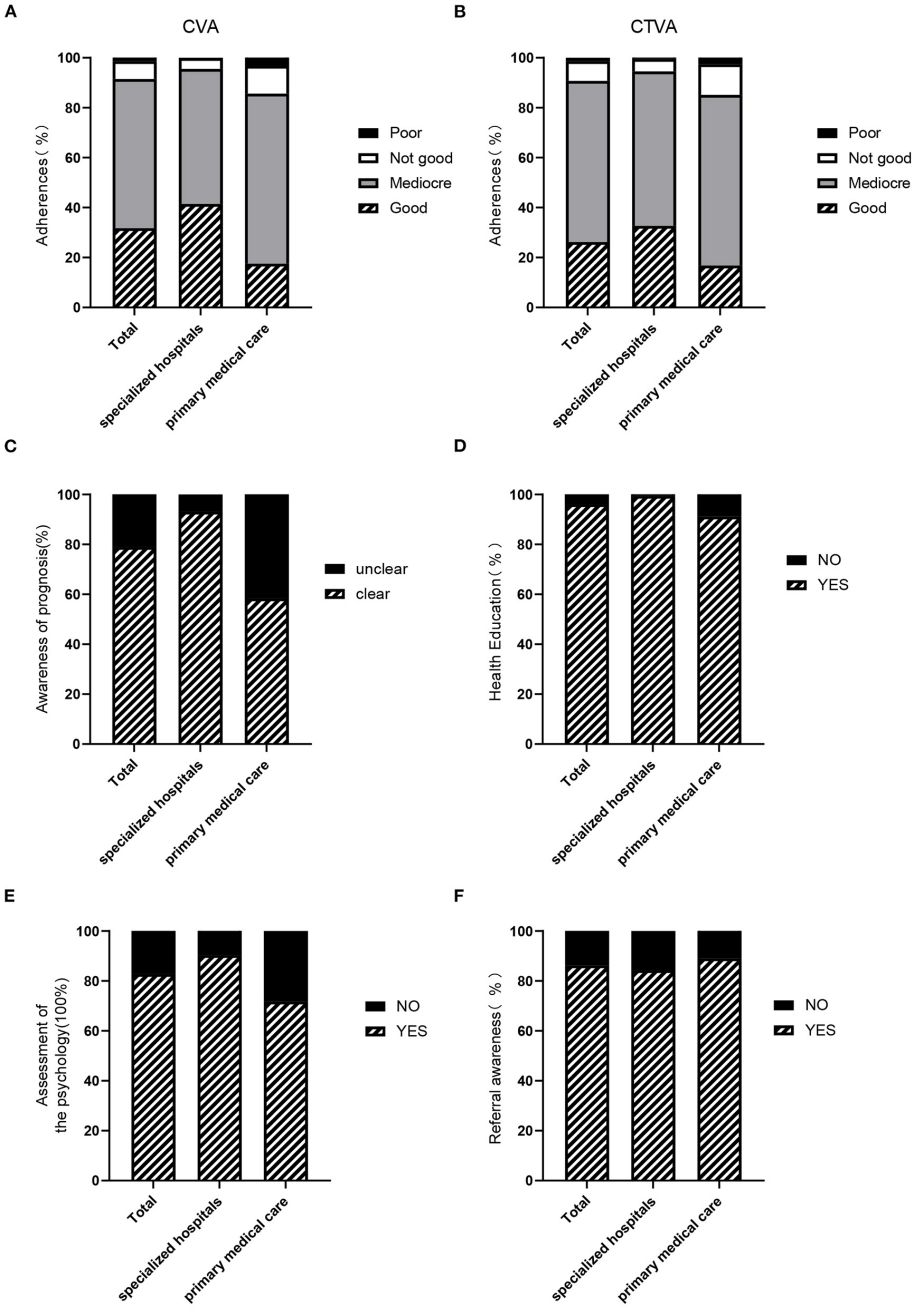
Education regarding and management of CVA and CTVA. **(A)** The compliance of patients with CVA in all facilities, specialized hospitals and primary medical care facilities. **(B)** Compliance of patients with CTVA in all facilities, specialized hospitals and primary medical care facilities. **(C)** Prognosis awareness of atypical asthma among physicians in all facilities, specialized hospitals, and primary medical care facilities. **(D)** Psychological assessments in patients with atypical asthma by physicians in specialized hospitals and primary medical care facilities. **(E)** Referral awareness of physicians in all facilities, specialized hospitals and primary medical care facilities. **(F)** Health education among patients with atypical asthma by physicians in all facilities, specialized hospitals, and primary medical care facilities.

Most physicians reported mediocre adherence in patients with CVA (59.75%, Figure 4A and Table 4). A similar result was also observed in patients with CTVA (64.48%, Figure 4B and Table 4).

**Table 4:** Adherences in patients with CVA and CTVA.

	**Adherence**	**Total**	**Specialized hospitals**	**Primary medical care**	*z* value^*^	***P*-value**
CVA	Good	623 (31.70%)	483 (41.42%)	140 (17.52%)	11.79	<0.0001
	Mediocre	1,174 (59.75%)	630 (54.03%)	544 (68.09%)		
	Not good	140 (7.13%)	51 (4.37%)	89 (11.14%)		
	Poor	28 (1.42%)	2 (0.17%)	26 (3.25%)		
CTVA	Good	515 (26.21%)	381 (32.68%)	134 (16.77%)	9.3	<0.0001
	Mediocre	1,267 (64.48%)	721 (61.84%)	546 (68.34%)		
	Not good	156 (7.94%)	58 (4.97%)	98 (12.27%)		
	Poor	27 (1.37%)	6 (0.51%)	21 (2.63%)		

* *Mann-Whiteney test*.

Of all participants, 1,551 (78.93%) physicians self-reported that they knew of the prognosis and outcome of atypical asthma clearly, among which, specialists were higher than PCPs (93.05% vs. 58.32%, *P* < 0.001). In other words, 333 (41.68%) PCPs were unclear and unsure (Figure 4C and Table 5).

**Table 5:** The education and management of atypical asthma.

		**Total**	**Specialized hospitals**	**Primary medical care**	***c*2 value**	***P*-value**
Awareness of prognosis	Clear	1,551 (78.93%)	1,085 (93.05%)	466 (58.32%)	343.88	<0.0001
	Unclear	414 (21.07%)	81 (6.95%)	333 (41.68%)		
Health Education	Yes	1,887 (96.03%)	1,159 (99.40%)	728 (91.11%)	85.39	<0.0001
	No	78 (3.97%)	7 (0.60%)	71 (8.89%)		
Assessment of the psychology	Yes	1,626 (82.75%)	1,053 (90.31%)	573 (71.71%)	114.82	<0.0001
	No	339 (17.25%)	113 (9.69%)	226 (28.29%)		
Referral awareness	Yes	1,694 (86.21%)	983 (84.31%)	711 (88.99%)	8.74	0.0031
	No	271 (13.79%)	183 (15.69%)	88 (11.01%)		

99.40% of specialists and 91.11% of PCPs reported that they would participate in a workshop to teach patients about atypical asthma (Figure 4D and Table 5).

90.31% of specialists and 71.71% of PCPs reported they assessed patients' mental health, respectively. In total, 82.75% of the physicians reported evaluating mental status (Figure 4E and Table 5).

Figure 4F shows that 86.21% of the physicians reported that they would refer patients with an unclear diagnosis or treatment failure to a different hospital. The rates of referral in primary care facilities and specialized hospitals ranged from 84.31% to 88.99% (Figure 4F and Table 5).

**Figure 5:**
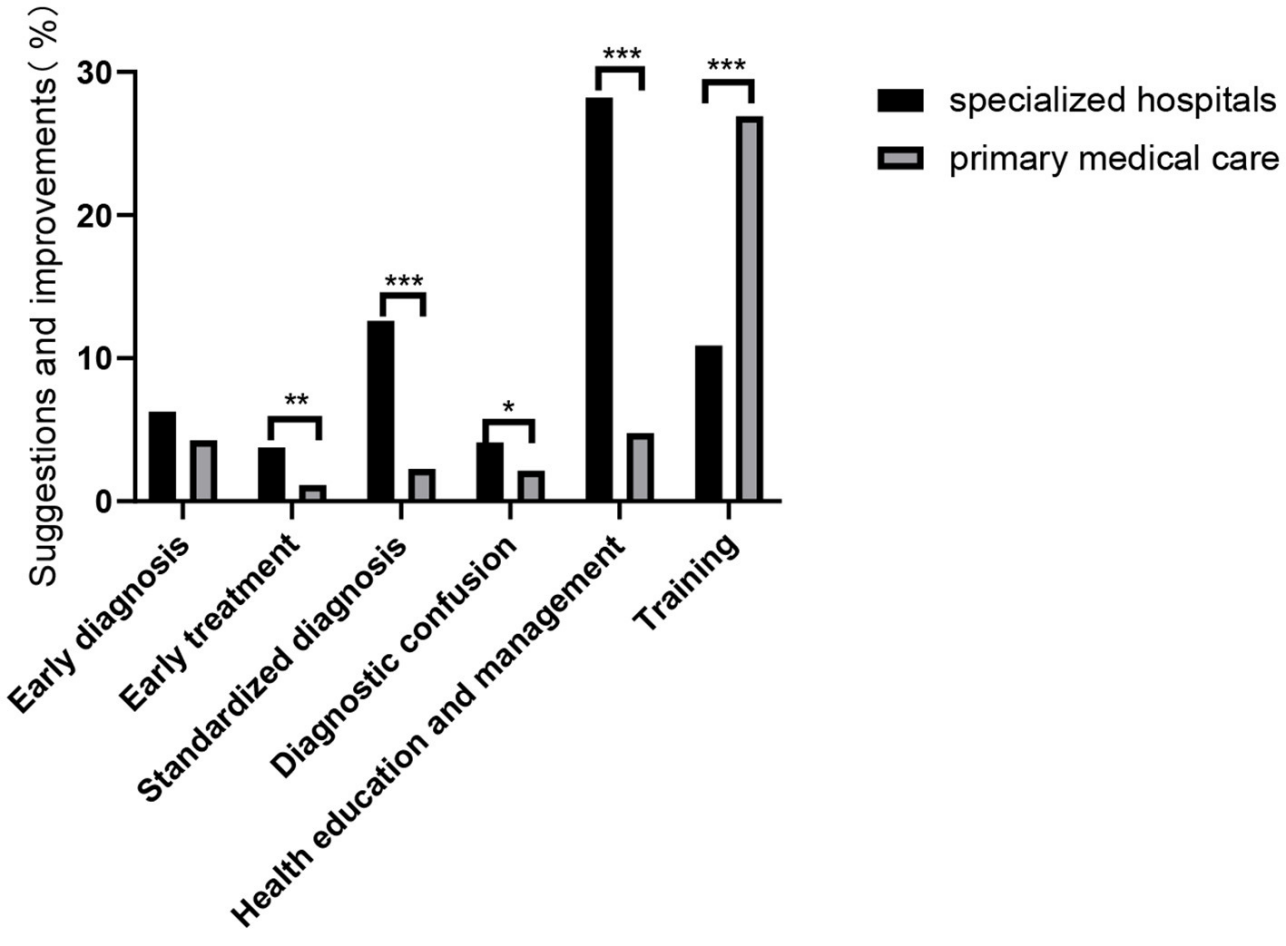
Education regarding and management of CVA and CTVA. Suggestions for improvements by physicians in specialized hospitals and primary medical care facilities. *P < 0.05, **P < 0.01, ***P < 0.001.

The most common suggestion and request by PCPs were for training and guidance (26.91%). Regarding specialists, the most common suggestions were to improve patient education and follow-up (28.22%, Figure 5).

## Discussion

This is the first large-sample survey on the knowledge and practices related to atypical asthma worldwide. Our present data showed that specialists reported higher awareness and better clinical habits than PCPs. Also it revealed that there were some defects in diagnosis and treatment among both specialists and PCPs.

Since 2016, CVA, CTVA and occult asthma have been discussed in asthma guideline in China (7, 8). Our data showed that specialists reported good awareness of CVA, followed by CTVA and occult asthma. The self-reported awareness of PCPs was relatively low, especially for CTVA and occult asthma. Compared to CVA, CTVA was a “young” disease, which was first reported by Shen et al. (6). Shen et al. further found that CTVA was associated with airway inflammation similar to that of typical asthma (22). A one year multicenter, prospective clinical study showed that patients with CTVA obtained efficacy by using the same treatment scheme (including ICS) recommended by the guidelines as typical asthma (12). Occult asthma emphasized people with asymptomatic bronchial hyperresponsiveness are at high risk of asthma (5, 23). This type of atypical asthma is far from clinical attention.

Physician's agreement with and adherence to the diagnostic criteria recommended by guidelines were assessed. The degree to which the diagnostic criteria were adopted by specialists was high. A total of 90.82% and 88.59% made correct diagnosis for CVA and CTVA, respectively. However, awareness of the diagnostic criteria among PCPs was remarkably lower (58.57% for CVA and 58.95% for CTVA). Previous analysis demonstrated that adherence to guidelines was higher among specialists than among PCPs (24–26). This was similar to the data obtained in our survey. Substantial financial investments have been made and infrastructure improvement has been achieved in primary medicine in the past decade in China; however, the quality of primary cares is still suboptimal, especially for diagnostic processes (27, 28). The prime problem in the primary cares is the lack of highly educated and trained physicians.

Our survey also found that there was an apparent disparity between awareness of and clinical practices for the diagnosis. The Expert Panel Report 3 (EPR 3) guideline states that “Spirometry is an essential objective measure to establish the diagnosis of asthma because the medical history and physical examination are not reliable means of excluding other diagnosis or for assessing lung states” (29). Therefore, physicians should seek objective evidence of variable airflow obstruction before making a diagnosis. While, low usage of spirometry, methacholine challenge test or PEF was found in this survey. Moreover, our survey showed that there was an obvious lack of onsite equipment. In total, 22.70% of hospitals did not conduct lung function, 70.08% did not have the ability to perform methacholine challenge tests, although which are important tests used in CVA and CTVA diagnosis.

Up to 95.88% and 96.14% of prescriptions by specialist included ICS or ICS/ LABA for the treatment of CVA and CTVA, while the corresponding values for prescriptions by PCPs were 85.60% and 85.98%, respectively. Moreover, an ICS alone or an ICS/LABA was prescribed by only a small proportion of physicians. A variety of other anti-asthmatic medicines or cough medicines, including theophylline, compound methoxyphenamine, traditional Chinese medicines, OCSs, and albuterol, were prescribed in combination with an ICS or an ICS/LABA for CVA or CTVA in most cases. However, it was previously reported that there was no clinical benefit to combine an inhaler with LTRAs, OCSs, or LTRAs plus an OCSs (30). This co-prescription meant increased medical expenses.

The recommended course of treatment for CVA is at least 8–12 weeks in the 2020 Chinese asthma guidelines (7) and 2015 Chinese cough guidelines (21). To date, there has been no standard for the course of treatment for CTVA. Our survey showed that the course of treatment in the real world was shorter than that recommended by the guidelines; this was very notable in primary care facilities. One of the reasons for this identified in our survey was that only 48.1% of primary care facilities had ICSs and LABAs in their pharmacies.

This study reports the results of the first online questionnaire conducted *via* WeChat about atypical asthma. This method was highly efficient and achieved a higher response rate than a previous traditional survey with a paper questionnaire (31). However, our study still had some limitations. First, the questionnaire was filled by participants themselves, so the quality of the data was not as good as these exported from EMRs system. Second, young people are still the predominant group using WeChat. Our study might have omitted a very small number of participants (for example, physicians old than 60 years old), especially in primary care facilities. Third, the questionnaire was sent to primary care doctors *via* WeChat, which meant that PCPs had a social or professional relationship with the sender. Accordingly, these PCPs may have more respiratory knowledge than other general practitioners.

## Conclusion

Our survey demonstrated that atypical asthma, such as CVA, CTVA, and occult asthma, is well-known and is diagnosed, treated, and managed by specialists and some PCPs. However, this survey demonstrated that variation exists among different groups. Outstandingly, PCPs showed low adherence to diagnostic methods, recommended medication, and courses. Updating the guideline and training physicians especially toward PCPs in the future should improve atypical asthma outcomes.

## Data Availability Statement

The raw data supporting the conclusions of this article will be made available by the authors, without undue reservation.

## Ethics Statement

The authors obtained approval from the Ethics Committee of the Second Affiliated Hospital, Zhejiang University School of Medicine.

## Author Contributions

HH, WH, and RC analyzed the data, interpreted the results, and wrote the paper. YH and SY performed the investigation. CC, MZ, KH, HL, HS, and KL designed the study and edited the manuscript. All authors contributed to manuscript revision, read, and approved the submitted version.

## Conflict of Interest

The authors declare that the research was conducted in the absence of any commercial or financial relationships that could be construed as a potential conflict of interest.

## Publisher's Note

All claims expressed in this article are solely those of the authors and do not necessarily represent those of their affiliated organizations, or those of the publisher, the editors and the reviewers. Any product that may be evaluated in this article, or claim that may be made by its manufacturer, is not guaranteed or endorsed by the publisher.
