# Supplementary material for: Perspectives and Management of Atypical Asthma in Chinese Specialists and Primary Care Practitioners—A Nationwide Questionnaire Survey
Source: Front Med (Lausanne). 2021 Oct 21;8:727381. doi: 10.3389/fmed.2021.727381 (PMC8582351; doi:10.3389/fmed.2021.727381)
Supplement: Supplementary file 1 [file Table_1.DOCX]

**Table E1 Questionnaire on cognition, diagnosis and treatment of atypical asthma**

| Q1 | Do you know any atypical asthma? (multiple choice)  □ cough variant asthma (CVA) □ chest tightness variant asthma (CTVA) □ occult asthma □ other |
| --- | --- |
| Q2 | How do you know the above atypical asthma?  □ asthma guidelines □ cough guideline □ textbook □ literature □ WeChat and other new media □ other |
| Q3 | How many cases of CVA do you diagnose each year? |
| Q4 | How many cases of CTVA do you diagnose each year? |
| Q5 | When did you start diagnosing patients with CTVA? |
| Q6 | What do you think is the main basis for CVA diagnosis? (multiple choice)  □ Cough is the only or main symptom, without wheezing, shortness of breath and other symptoms and signs of typical asthma.  □ At the same time, there is any examination to support reversible airflow limitation.  □ Except for other diseases that cause cough.  □ It is effective to treat disease according to treatment for typical asthma. |
| Q7 | What do you think is the main basis for CTVA diagnosis? (multiple choice)  □ Chest tightness is the only or main symptom, without wheezing, shortness of breath and other symptoms and signs of typical asthma.  □ At the same time, there is any examination to support reversible airflow limitation.  □ Except for other diseases that cause chest tightness.  □ It is effective to treat disease according to treatment for typical asthma. |
| Q8 | Is there any lung function instrument in your institution?  □ YES □ NO |
| Q9 | Is there bronchial provocation test in your institution?  □ YES □ NO |
| Q10 | Is there FeNO test in your institution?  □ YES □ NO |
| Q11 | Is there induced sputum test in your institution?  □ YES □ NO |
| Q12 | Which method would you choose to diagnose atypical asthma? (multiple choice)  □ Pulmonary ventilation function and bronchial dilation test  □ Pulmonary ventilation function and bronchial provocation test  □ PEF variation  □ FeNO  □ Induced sputum  □ Inquiry of medical history  □ Asthma treatment regimens are effective. |
| Q13 | What is your first choice for a newly diagnosed CVA? (multiple choice)  □ ICS □ ICS/LABA □ Compound Methoxyphenamine Capsules □ Oral corticosteroids □ Montelukast □ Theophylline □ Salbutamol □ Antimicrobial □ Chinese traditional medicine □ Other |
| Q14 | What is your first choice for a newly diagnosed CTVA? (multiple choice)  □ ICS □ ICS/LABA □ Compound Methoxyphenamine Capsules □ Oral corticosteroids □ Montelukast □ Theophylline □ Salbutamol □ Antimicrobial □ Chinese traditional medicine □ Other |
| Q15 | What is the treatment outcome for patients with CVA in your care?  □ very good □ in general □ not good □ poor |
| Q16 | What is the treatment outcome for patients with CTVA in your care?  □ very good □ in general □ not good □ poor |
| Q17 | Do you pay attention to and evaluate the mental health status of patients with atypical asthma?  □ YES □ NO |
| Q18 | Would you recommend a transfer treatment for patients with atypical asthma who are not responding well to treatment?  □ YES □ NO |
| Q19 | Do you know the outcome and prognosis of atypical asthma?  □ very clear □ clear □ not quite clear □ no |
| Q20 | Do you provide health education for patients with atypical asthma?  □ very detailed □ in general □ basically no □ no |
| Q21 | Do you have any suggestions on the diagnosis and treatment of atypical asthma? |
